# The Role of Burnout in the Association between Work-Related Factors and Perceived Errors in Clinical Practice among Spanish Residents

**DOI:** 10.3390/ijerph18094931

**Published:** 2021-05-06

**Authors:** Isabel Saavedra Rionda, Laura Cortés-García, María de la Villa Moral Jiménez

**Affiliations:** 1 Mental Health Unit of the Health Service of Asturias, 33800 Asturias, Spain; 2 PROMENTA Research Center, Department of Psychology, University of Oslo, 0373 Oslo, Norway; l.c.garcia@psykologi.uio.no; 3 Section of Social Psychology, Department of Psychology, Faculty of Psychology, University of Oviedo, 33003 Oviedo, Spain; mvilla@uniovi.es

**Keywords:** burnout, work-related factors, perceived errors, residents, mediation model

## Abstract

This study aimed to evaluate the prevalence of burnout syndrome in a sample of residents from different specialties, to determine the influence of work-related factors on the development of burnout, and to examine the mediating role of burnout in the relation between such work factors and perceived errors in clinical practice. A total of 237 Spanish residents participated (*M_age_* = 28.87, *SD* = 3.84; 73.8% females). The Maslach Burnout Inventory and an ad hoc questionnaire were administered to assess burnout and work-related factors. Comparison analyses and mediational models were conducted. Half of the residents reported high levels of burnout (48.9%). Burnout was significantly associated with perceived errors in clinical practice. Significant differences were found between residents with lower and higher burnout levels, showing that those with higher burnout were less satisfied with the working conditions. Burnout mediated the associations between adjustment of responsibility, support among residents, satisfaction with teaching and rotations, general satisfaction, and perceived errors in the clinical practice. Adjusted levels of responsibility and workload, enhanced supervision, and more social support from colleagues predict lower levels of burnout, which may result in fewer errors in clinical practice. Consequently, such work-related factors should be taken into account as a preventive strategy for burnout and errors in the clinical practice so adequate patient care, good mental health of future specialists, and, therefore, higher quality of public health care can be ensured.

## 1. Introduction

Burnout is a syndrome that involves emotional exhaustion, depersonalization and a decrease in self-fulfillment, which is caused by constant involvement in emotionally demanding situations [1,2]. Residents are an especially vulnerable group to suffer from burnout, mainly because of the work surroundings, where they have to face with many situations of suffering, pain and death, where the constant patient contact demands a high level of engagement and a great responsibility, and where decisions must sometimes be made on an urgent basis [3]. Moreover, residency is a period of transition and uncertainty, where residents must confront new situations in which the burden of care and the degree of demands imposed may not match the year of residency [4]. In this respect, research shows that residents present even higher levels of burnout than medical specialists with more years of experience [5]. Accordingly, the prevalence of burnout syndrome among residents fluctuates between 20% and 60%, which implies a significant negative impact not only on their wellbeing but on the quality of the care provided [6].

Three categories of risk factors associated with burnout syndrome have been noted: sociodemographic, psychological and work-related [7]. Although no consensus has been reached regarding the influence of these factors on the development of burnout, according to Maslach’s theory, burnout syndrome is mainly associated with work-related aspects. More concretely, the most relevant work-related aspects involve: (a) overwork, that is, an elevated number of hours and lack of rest (e.g., failure to schedule rest periods after on-call shifts) and the need to continually update knowledge, among others; (b) lack of control over the tasks themselves and the physical work environment, low involvement in decision-making and scarcity of technological resources, etc.; (c) insufficient recognition for and feedback on the work accomplished; (d) feelings of unfairness, that is, unsatisfactory economic conditions and lack of future perspectives and occupational growth; (e) structural errors when designing a workplace community (e.g., lack of spaces for dealing with difficulties in interpersonal relationships); (f) conflict of values between personal values and those of the organization [8].

An increasing number of studies underscore the importance of exploring how different work-related factors are associated with the levels of burnout among residents, especially because such knowledge would inform preventive efforts [9,10]. Among the work-related factors associated with burnout, overwork and few hours of sleep [11,12], together with lacking communication, poor relationships among peers and inefficient organizational and supervisory aspects [13,14,15] have been identified as the most significant. The relevance of the analysis of the work-related factors lies in the empirical evidence of the consequences of burnout, not only because of its impact on the residents’ health, but also on the quality of care that the residents provide to the patients, which might be deteriorated [16]. As such, one of the main focuses of recent studies in the field has been understanding how burnout levels relate to errors in the clinical practice of residents [17,18]. In this regard, it has been found that burnout indeed precipitates more errors and reduces the quality of medical services [19]; however, it is still unclear whether it is the perception of committing more errors in clinical practice that is greater among residents with a high degree of burnout or whether they actually commit these errors [20]. Relatedly, previous studies have shown that residents with the highest level of working stress tend to be more self-critical and, therefore, more inclined to report having committed errors [21,22]. According to Firth-Cozens [21], it is important to emphasize that even health providers with good physical and mental health commit errors in their daily work and are not free of feelings of distress or worry. It is in fact when professionals suffer from burnout syndrome that they are more vulnerable to develop anxiety or depression [23], and therefore, when they are at greater risk of committing errors in their clinical practice [24].

In order to address burnout syndrome, it is crucial not only to identify risk factors [8], but also those factors that may be considered as protective [25]. For instance, work-related factors that could contribute to the residents’ satisfaction include the role played by the supervisor [26]. Residents’ supervisors support their trainees and address their difficulties and concerns, especially when they face challenging situations at work. Moreover, supervisors promote the acquisition of emotional skills among residents [27]. There is evidence that those residents who benefit from an optimal supervision and support across the residency period display a lower level of emotional exhaustion and depersonalization than those who do not receive adequate support from their supervisors [28]. In this respect, one of the goals of training as a resident is to gradually increase autonomy and control over decisions, although it has been demonstrated that autonomy and assumption of more and more responsibilities can be a major source of stress if these levels exceed their abilities for managing and coping with them [29]. In other words, another important area in which the supervisor must be involved is determining that the level of autonomy and responsibility acquired is appropriate for the residents’ skills and for the year of residency [29]. Likewise, it has been demonstrated that a positive training environment has a direct influence on reducing levels of burnout, and it has also been observed that it is indirectly related with better teamwork, collaboration among peers and better quality of provided care to patients [16]. Accordingly, it has been found that wellbeing, self-confidence and skills of residents improve when they receive adequate supervision, which, in turn, would be reflected in their clinical practice, contributing to a better quality of care and reduction of errors [30].

In addition, a positive working environment—creating a collaborative atmosphere both with other residents and with the rest of the professionals—constitutes an important factor in protecting residents from burnout that may increase the level of wellbeing and lead to committing fewer errors in provision of care, thereby resulting in improved patient care [30,31]. Concretely, there is evidence indicating that providing a positive working atmosphere—including support among residents, an adequate workload matching their skill levels and sensitive supervisors that they can turn to in the presence of complicated situations, doubts, or distress—are associated with improved quality of life, greater job satisfaction and less burnout [32,33].

Taken together, there is robust support on the potential influence that different work-related factors have on the level of burnout in residents and in decreasing or increasing perceived errors in the clinical practice; however, so far, no study has elucidated the interplay between these variables among Spanish residents. Consequently, the present study aims to (1) examine the level of burnout syndrome among residents from different specialties; (2) determine the influence of various work-related factors on the development of burnout syndrome; (3) explore the associations between work-related factors, burnout and perceived errors in clinical practice; (4) analyze the mediating role of burnout in the relationship between work-related factors and perceived errors. In line with theory and previous studies, we expected to find significantly high levels of burnout among residents across the different specialties, and that work-related factors will influence the levels of burnout. We also hypothesized that work-related factors, burnout and perceived errors in clinical practice will be associated. Regarding the mediational role of burnout, based on previous studies supporting the association between the variables of interest, we expected that the level of burnout could explain, at least partly, the association between work-related factors and perceived errors in the clinical practice; however, because this is the first study testing burnout as mediator, we did not make more specific hypotheses.

## 2. Materials and Methods

### 2.1. Participants

This study is part of a more extensive project on burnout among residents. All residents from all specialties, from the first to the final year of residency at two Spanish hospitals were invited to collaborate. A total of 952 residents were invited to participate. Of these, 237 returned completed surveys, so they were included for the final sample for the present study. The majority of them were women (73.8%), with an average age of 28.87 (*SD* = 3.84) and an age range between 25 and 32 years.

### 2.2. Instruments

The *Maslach Burnout Inventory (MBI)* was used to measure burnout, since it is the reference survey for determining the degree of perceived burnout. It consists of 22 items measured using a five-point Likert scale (where 1 represents a response level of never, and 5, every day). It is made up of three subscales: Emotional exhaustion, Depersonalization and Personal accomplishment. For correction, the following cut-off points were utilized: for emotional exhaustion, a score above 27 was considered to indicate a high level of burnout, a score between 19 and 26, a medium level, and a score below 19, a low level; in the depersonalization subscale, scores above 10 were taken to indicate a high level of burnout, between 6 and 9 a medium level of burnout and scores lower than 5, a low level of burnout; lastly, on the personal accomplishment subscale, where scores were given inversely, high personal accomplishment was considered a score greater than 40, medium, a score from 34 to 39 and low, a score less than 34. For correction, independent scores were derived for the different subscales, and high burnout was considered to apply to those who received high scores in emotional exhaustion and/or in depersonalization, together with a low or medium score in personal accomplishment. The remaining combinations of scores were considered cases of low burnout. Cronbach’s alphas obtained for burnout, emotional exhaustion, depersonalization, and personal accomplishment were 0.75, 0.89, 0.76, and 0.82, respectively.

To measure the work-related factors of interest for this study, an ad hoc questionnaire (α = 0.93) was designed. This instrument includes 38 items that focus on work-related factors that are specific to the training period of residency. In this questionnaire the resident is asked about their level of agreement with statements related with the quality of supervision provided by the supervisor(s) (8 items), adjustment of responsibility to the skills acquired in each year of residency (9 items), information and satisfaction with the teaching and rotations (4 items), opportunities for research (3 items), working atmosphere (4 items), support among residents (4 items) and satisfaction with the training, specialty and location selected to pursue it (6 items). Some examples of these items are: “I have too much workload”, “My service works as a team” or “I can rely on other residents when I need help”. Likewise, nine items were included regarding errors and attitudes toward patients previously used in another study [34]. A Likert scale was used from 1 (never) to 5 (always). The higher the score, the greater the magnitude of the variable studied. The theoretical average of each group was calculated (total of the minimum and maximum possible score for each item divided by the number of items in each section) as a point of reference for the deviation of the real average score derived from the responses of the participants.

### 2.3. Procedure

All participants received an email explaining the aim of the study and inviting them to fill in an online survey on a voluntary basis, ensuring the confidentiality of the data. The online survey included sociodemographic data such as gender, age, specialty, year of residency, living arrangements, on-call shifts worked and scheduling of rest periods after on-call shifts, together with the rest of assessment measures. The survey, which took roughly 20 min to complete, was available to residents for 4 months (i.e., from December 2018 to March 2019). Participation was voluntary, anonymous and confidential. This study was approved by the Ethics Committee of Research of the Principality of [blinded for review] (Spain).

### 2.4. Data Analysis

Data analyses were carried out using R program, version 3.6.0 (R Foundation for Statistical Computing, Vienna, Austria) [35,36]. Descriptive analyses of sociodemographic variables and the variables of interest were conducted. Comparative analyses were performed in order to determine whether work-related factors varied according to the levels of burnout among residents. Concretely, we performed χ^2^ tests and independent sample t tests to examine associations between burnout levels and demographic characteristics (age, gender, origin, marital status), prior specialty, on-call shifts, and scheduling of rest periods between on-call shifts. We also performed a t test to examine differences in work-related factors and errors according to the level of burnout. Then, we ran ANOVAs to compare groups by specialty (i.e., medical, surgical and M-S, laboratory, metal health, nurse, and others) and by year of residency on burnout levels. Significant ANOVAs were followed up using Tukey’s test to control for multiple comparisons. Correlation analyses were also run including burnout, work-related factors and perceived errors in clinical practice. Lastly, mediation analyses were conducted, in which work-related factors were the independent variables, burnout the mediator and perceived errors the dependent variable. Sobel test was used to calculate the mediating effect size. The significance level utilized was 0.05.

## 3. Results

### 3.1. Characteristics of the Sample

As seen in Table 1, more than half of the responses were from first-year and second-year residents (60%). Half of the participants were residents of a medical specialty; the other 50% was distributed among specialties of mental health, nursing, or pharmacy, among others. Of those who answered (all specialties, except psychologists and nurses working on mental health services) that they worked on-call shifts (91.1%), only 54.9% had rest periods scheduled between shifts. In responding whether they had completed a specialty previously, the majority of residents (96.6%) reported being trainees in their first specialty. In total, 58.2% were from the city where the study was conducted, and the rest came from a different Spanish autonomous community (32.1%) or from another country (9.7%). Finally, 88.2% of the residents were married, and the rest were distributed among married/domestic partner (9.7%), separated/divorced (1.7%) and widowed (0.4%).

Regarding burnout, almost half of the residents reported a high level of burnout (N = 116, M = 65.91, SD = 9.58). The mean scores of each subscale were as follows: 25.63 for emotional exhaustion (medium burnout), 10.88 for depersonalization (high burnout) and 29.4 for personal accomplishment (high burnout). More specifically, 44.7% of the residents registered a high score in emotional exhaustion, 53.6%, a high score in depersonalization and 74.7% reported low personal accomplishment.

### 3.2. Group Differences

No statistically significant differences were detected in the level of burnout by gender, age, scheduling of rest periods between on-call shifts, prior specialty, origin and marital status. On the contrary, there were significant differences related to working on-call shifts, the specialty and the year of residency. Concretely, higher level of burnout was found among those who worked on-call shifts (χ^2^ (1, *N* = 216) = 8.24, *p* = 0.004). Additionally, those who were in the last years of training had higher levels of burnout (see Table 2). Regarding the specialty, those who were trained in medical specialties reported higher levels of burnout (see Table 3). Interestingly, post hoc comparisons indicated that mean score in burnout for the surgical specialties (*N* = 41, M = 70.98, SD = 8.88) was significantly different than medical specialties (*N* = 120, M = 66.46, SD = 10.43). However, laboratory specialties (*N* = 5, M = 59.8, SD = 6.18) did not significantly differ from the surgical specialties.

Table 4 shows that there were statistically significant differences in all work-related factors studied by the degree of burnout, since the mean score of each of the factors was greater in the low-level burnout group, indicating that higher scores in each of the work-related factors are found among those with low level of burnout. Likewise, significant differences were observed among groups regarding perceived errors. Concretely, more perceived errors were reported among those with high levels of burnout.

### 3.3. Bivariate Relations between Main Study Variables

Table 5 shows the results from correlation analyses. Work-related factors were negatively correlated with burnout. Perceived errors, on the other hand, correlated positively with burnout and negatively with all work-related factors. The associations between the different work-related factors were positive in all cases.

### 3.4. Mediation Analysis

Table 6 shows the results of the mediation analyses. Concretely, Sobel tests proved that burnout fully mediated the association between support among residents (*z* = −2.25, *p* = 0.025), satisfaction with the specialty (*z* = −2.46, *p* = 0.014) and the perceived errors. Burnout partly mediated the associations between teaching and rotations (*z* = −2.27, *p* = 0.023), adjustment of responsibility (*z* = −1.96, *p* = 0.049) and perceived errors. In the case of supervision (*z* = −1.88, *p* = 0.061) and work atmosphere (*z* = −1.70, *p* = 0.089), there was no mediation effect. Mediation was also ruled out for the factor of research (*z* = −1.63, *p* = 0.101).

## 4. Discussion

Broad research has demonstrated that the prevalence of burnout syndrome and its negative consequences among healthcare professionals and, concretely, among residents, are high [13,37,38,39,40]. In recent years, the main focus of studies on burnout among residents has been determining work-related factors that could contribute to improve the working environment of the residents, which would result in an important reduction in the levels of burnout, in errors in the clinical practice and, therefore, in an improvement in the quality of care [13,15,41,42]. According to Zutaitiené et al. [15], some of the work-related factors that have been identified as the most influential in the development of burnout syndrome and subsequent errors in the clinical practice are elevated pressure to provide care, lack of job security, lack of supervision and support among coworkers. To date though, no studies have elucidated the interplay between such variables among Spanish residents from different specialties. To fulfill this need in the field of care providers’ wellbeing and public health investigation, the present study was conducted. In line with previous literature, we found that indeed such work-related factors are associated with more burnout syndrome and perceived errors in the clinical practice. Moreover, our mediational model expands such knowledge by elucidating that the indirect effect of work-related factors on the development of perceived errors in the clinical daily practice of residents is exerted via higher levels of burnout syndrome.

In the current study, residents showed high levels of burnout, both in general and on each subscale, showing a high degree of emotional exhaustion and depersonalization and a low degree of personal accomplishment, which aligns with previous research [4]. High level of burnout was not associated with sociodemographic variables such as gender, age, origin or marital status [43], but it was associated with work-related factors that depend on the structure and culture of the organization, as expected [19,41]. First, those who worked on-call shifts presented higher levels of burnout, which is in congruence with studies indicating that call-on shift workers may develop burnout syndrome due to, at least partly, the rapid response and efficiency necessary to save lives in emergencies, fatigue or lack of sleep and the legal and moral responsibility in decision making [44,45]. Second, the medical specialties and more specifically, surgical medical specialties were the most affected [46] possibly related to the stressful situations and increased daily risk involved in the surgical interventions they have to face [47]. Lastly, residents in the last years of training showed higher levels of burnout, which indicates that as the residency period progresses, residents may be more vulnerable to develop burnout syndrome, especially when specific work-related conditions are unsatisfactory across time. Such findings call for the need to implement preventive programs during the residency period to reduce the risk to develop burnout syndrome [48]. Overall, these findings are consistent with the fact that the residency constitutes a transitional period that involves a complex learning process, in which, it is necessary to take into account that the resident confronts in a rather abruptly way, stressful situations in a daily basis, assumes a high level of responsibility and is subject to other organizational changes that are prolonged across the four or five years of the residency period. In this context, residents’ perceptions about how the institutions deal with these aspects are associated with their level of burnout [49]. Therefore, it is necessary to consider the development of personal skills such as the search for autonomy and an own judgment, the intervention on possible emotional maladjustments, as well as the optimization of coping skills and mechanisms that might help in their adaption to the high-demanding environment during the residency period. Such interventions will contribute to the prevention of burnout syndrome among residents and will ensure an optimal patient care [29,50,51].

Related to this, our results showed that there were differences in the influence of work-related factors between the two groups of burnout levels (i.e., high vs. low). Concretely, residents with a lower level of burnout reported having approachable supervisors to which they could rely on, having a level of responsibility appropriate for their level of training, being satisfied with the teaching and the rotations, as well as with opportunities for research; they also reported having a pleasant work atmosphere, feeling supported by their peers and satisfied with the specialty and hospital selected to pursue the residency. Hence, as expected, our results agree with the research conducted up to date regarding the importance of taking into account all these aspects related with the environment and quality of learning [27,52]. In fact, it has been demonstrated that successful supervision can aid in early identification of burnout, thereby promoting the development of adequate coping strategies among residents [53]. In addition, there were significant differences in terms of perceived errors between the groups of burnout. Concretely, the group showing higher levels of burnout also perceived poor quality of patient care and more perceived errors in their clinical practice. Such results confirm our hypothesis, and pinpoint to the fact that patients treated by residents who are experiencing burnout might have a higher risk of receiving a lower quality of care [54]. Our findings highlight the importance of promoting adequate supervision and working conditions during residency that hinder the appearance of burnout among residents and, therefore, may reduce the probability of committing errors in clinical practice [55].

The present investigation found significant negative associations between all work factors studied and burnout levels. Such findings confirm the importance of promoting consistent supervision, providing a collaborative and supportive atmosphere, facilitating progressive acquisition of autonomy and feelings of control and security to reduce the risk for developing burnout syndrome [16]. These results suggest that the more these organizational strategies are designed and implemented, the lower the level of burnout perceived and the fewer the errors; in other words, attention to these aspects implies promoting an adequate skill level, including emotional skills, reflection, critical thinking and values which will enable residents to cope more effectively with the difficulties of their daily work [27,29].

Our mediational model demonstrated that burnout mediated among almost all work-related factors studied and perceived errors. Concretely, the indirect effect of poor teaching and rotations and lack of adjustment of responsibility on higher perception of errors in the clinical practice was exerted via burnout. However, such mediating effect was partial, which suggests that there might be other aspects that may explain such relationship that need to be further studied. There was, nonetheless, complete mediation in the case of low satisfaction with the specialty and support among residents, which indicates that the influence of these variables on more perceived errors might be explained by the detrimental effect of burnout. Although no mediational effects of burnout were found in the models including supervision and the work atmosphere as predictors, previous research has demonstrated their crucial role for the early detection of burnout [29]. Likewise, the influence of variables such as workload and working hours, sleep deprivation, attention to personal life, scheduling rest periods after on-call shifts and feedback for the residents should not be overlooked [29,56,57,58]; these factors should be examined together with the variables from this study in future research in order to promote more comprehensive workplace policies that allow a better management of the crucial period of residency.

Taken together, our results indicate that the training of specialists constitutes a great challenge for the institutions, since they have to manage many different work-related factors in order to reduce the risk for the development of burnout and perceived errors in clinical practice. Specifically, based on our findings, it is recommendable to include specific training programs for residents’ supervisors, coupled with the support of the organization on implementing specific programs that address the different needs of residents in terms of academic training and development of critical skills [15]. Hence, this study paves the way for more studies that will treat in greater depth the specific training of the supervisors [29], the availability of structured supervision programs and the evaluation of these programs [53], the influence of different work-related factors in development of public and work-related policies for healthcare professionals [55], the development of tools to prevent errors and the establishment of a culture and workplace community that accepts imperfections in individual performance in order to promote turning to supervisors and peers when confronted by the dilemmas and difficulties inherent in the profession [18]. Therefore, the ultimate goal is for residents to profit from professional and personal support from the institution that encourage different initiatives to reduce burnout and to improve quality of care [42,53,58].

### Limitations and Strengths

The current results should be interpreted in the context of some limitations. First, the low response rate made it difficult to draw a general conclusion from the results. Such low response rate is, however, common in studies that use online questionnaires [59]. Relatedly, our results must be viewed with caution and cannot be generalized to all Spanish residents. Second, a random sample was not taken since the survey was aimed at the entire sample, and it would be necessary to analyze the bias derived from the response rate. Third, our results could not indicate causality between the variables due to the cross-sectional design of the study. Lastly, the influence of other variables that could interfere with the levels of burnout, and also in the perception of errors, could not be controlled for. Despite the aforementioned limitations, the current study provides novel results in this field of research and presents important strengths including a more in-depth approach on crucial work-related factors that influence the wellbeing of Spanish residents and that help in the prevention of errors in clinical practice.

## 5. Conclusions

The results of our study suggest that there are different factors associated with the working environment that should be addressed in order to mitigate the risk to develop burnout syndrome and improve the quality of the healthcare provided by residents. Accordingly, and especially during these difficult times related to the COVID-19 pandemic, primary prevention should be a top-priority goal for institutions that run programs for training specialists so that we not only train professionals with a high degree of wellbeing, but also satisfied patients and top-quality healthcare.

## Figures and Tables

**Table 1:** Participants’ sociodemographic characteristics and working conditions.

	*n*	%
**Sex**		
Females	175	73.8
Males	62	26.2
**Rest period between shift (*n* = 216)**		
Yes	130	54.9
Sometimes	46	19.4
No	40	16.9
**Residency’s year**		
First	70	29.5
Second	72	30.4
Third	42	17.7
Fourth	46	19.4
Fifth	7	3
**Specialty**		
Medical	120	50.63
Surgical and M-S	41	17.29
Laboratory	5	2.1
Mental Health	19	8
Nurse	40	16.9
Others (e.g., Biologist, Pharmacist, Chemist)	12	5.1

Note: M-S = Medical-Surgical.

**Table 2:** Group differences in burnout and its subscales and year of residency.

Variable	First (*N* = 70) M(SD)	Second (*N* = 72) M(SD)	Third (*N* = 42) M(SD)	Fourth (*N* = 46) M(SD)	Fifth (*N* = 7) M(SD)	*F*	*p*	*η^2^*	Significant Post-Hoc Comparisons
Burnout	61.64 (8.37)	65.74 (9.36)	67.45 (10.36)	70.87 (8.74)	68.43 (6.27)	7.743	0.000	0.118	Fourth > First; Third > First; Fourth > Second
Emotional exhaustion	21.59 (6.38)	26.13 (6.69)	27.62 (7.77)	28.96 (7.88)	27.14 (5.58)	9.392	0.000	0.139	Fourth > First; Third > First; Second > First;
Depersonalization	9.81 (3.33)	10.53 (3.65)	11.86 (4.93)	12.20 (4.67)	10.71 (3.25)	3.219	0.013	0.053	Fourth > First
Personal accomplishment	30.24 (5.51)	29.08 (5.10)	27.98 (5.44)	29.72 (5.83)	30.57 (1.27)	1.353	0.251	0.023	

Note: M = mean; SD = standard deviation.

**Table 3:** Group differences in burnout and its subscales and residency’s specialties.

Variable	Medical (*N* = 166) M(SD)	Mental Health (*N* = 19) M(SD)	Nurse (*N* = 40) M(SD)	Others (*N* = 12) M(SD)	*F*	*p*	*η^2^*	Significant Post-Hoc Comparisons
Burnout	67.37 (10.19)	65.21 (6.79)	61.58 (6.89)	61.17 (6.30)	5.308	0.001	0.064	Medical > Nurse
Emotional exhaustion	26.67 (7.94)	24.21 (5.59)	22.43 (5.96)	24.08 (6.11)	4.049	0.008	0.050	Medical > Nurse
Depersonalization	11.66 (4.27)	8.95 (2.85)	9.03 (2.91)	9.33 (3.47)	7.266	0.000	0.086	Medical > Mental Health > Nurse
Personal accomplishment	29.04 (5.49)	32.05 (3.77)	30.13 (5.10)	27.75 (5.95)	2.445	0.065	0.031	

Note: M = mean; SD = standard deviation. Others = Biologists, Pharmacists, Chemists.

**Table 4:** Differences in work-related factors and errors according to the level of burnout.

	Burnout	M (SD)	*p*	Cohen’s *d*
Supervision	High	27.55 (7.42)	<0.001	0.57
	Low	31.50 (6.44)		
Adjustment of responsibility	High	28.89 (4.15)	<0.001	0.58
	Low	31 (3.07)		
Teaching and rotations	High	11.65 (3.29)	<0.001	0.67
	Low	13.75 (3.03)		
Research opportunities	High	8.53 (3.17)	0.004	0.38
	Low	9.70 (3)		
Work environment	High	13.69 (3.57)	<0.001	0.67
	Low	15.91 (3.09)		
Social support from other residents	High	15.23 (2.97)	<0.001	0.69
	Low	17.05 (2.3)		
Satisfaction with residency program	High	19.97 (4.25)	<0.001	0.73
	Low	22.93 (3.82)		
Perceived errors	High	19.16 (4.75)	<0.001	0.63
	Low	16.37 (4.11)		

**Table 5:** Results of the correlations between burnout, work-related factors and perceived errors.

	1	2	3	4	5	6	7	8	9
1. Burnout	-								
2. Supervision	−0.20 **	-							
3. Responsibility	−0.16 *	0.38 **	-						
4.Teaching and rotations	−0.27 **	0.53 **	0.47 **	-					
5. Research	−0.13 *	0.39 **	0.21 **	0.39 **	-				
6. Environment	−0.17 **	0.53 **	0.31 **	0.50 **	0.48 **	-			
7. Support	−0.26 **	0.29 **	0.16 *	0.27 **	0.29 **	0.50 **	-		
8. Satisfaction	−0.30 **	0.49 **	0.37 **	0.63 **	0.44 **	0.63 **	0.37 **	-	
9. Perceived errors	0.25 **	−0.24 **	−0.28 **	−0.25 **	−0.16 *	−0.21 **	−0.20 **	−0.17 *	-

** *p* < 0.01; * *p* < 0.05.

**Table 6:** Results of mediational analyses of the effect of working factors on perceived errors via burnout.

Independent Variable Mediator	X→Y		X→M		M→Y		R^2^
	β(SE)	CI	β(SE)	CI	β(SE)	CI	
Supervision							
Burnout	−0.14 (0.04)	[−0.22, 0.06]	−0.20 (0.09)	[−0.36, −0.03]	0.10(0.03)	[0.04,0.16]	0.022
**Adjustment of responsibility**							
Burnout	−0.26 (0.08)	[−0.41, −0.11]	−0.40 (0.16)	[−0.72, −0.08]	0.10(0.03)	[0.04, 0.16]	0.101
**Teaching and rotations**							
Burnout	−0.31 (0.09)	[−0.48, −0.13]	−0.64 (0.18)	[−0.99, −0.28]	0.09(0.03)	[0.03, 0.15]	0.103
Research opportunities							
Burnout	−0.22 (0.09)	[−0.40, −0.04]	−0.37 (0.20)	[−0.75, 0.02]	0.11(0.03)	[0.05, 0.17]	0.079
Work environment							
Burnout	−0.21 (0.08)	[−0.37, −0.05]	−0.34 (0.18)	[−0.69, 0.00]	0.11(0.03)	[0.05, 0.16]	0.082
**Social support from other residents**							
Burnout	−0.20 (0.11)	[−0.41, 0.01]	−0.66 (0.22)	[−1.09, −0.24]	0.10(0.03)	[0.04, 0.16]	0.038
**Satisfaction with the residency program**							
Burnout	−0.09 (0.07)	[−0.22, 0.05]	−0.51 (0.15)	[−0.78, −0.23]	0.11 (0.03)	[0.04, 0.17]	0.063

Note: Significant effects in bold. X = independent variable; M = mediator; Y = dependent variable.

## Data Availability

The data presented in this study are available on request from the corresponding author.
